# Computer Model-Driven Design in Cardiovascular Regenerative Medicine

**DOI:** 10.1007/s10439-022-03037-5

**Published:** 2022-08-16

**Authors:** Sandra Loerakker, Jay D. Humphrey

**Affiliations:** 1 Department of Biomedical Engineering and Institute for Complex Molecular Systems, Eindhoven University of Technology, Eindhoven, Netherlands; 2 Department of Biomedical Engineering and Vascular Biology & Therapeutics Program, Yale University and Yale School of Medicine, New Haven, CT USA

**Keywords:** Tissue engineering, Growth and remodeling, Finite element, Mechanobiology, Homeostasis

## Abstract

Continuing advances in genomics, molecular and cellular mechanobiology and immunobiology, including transcriptomics and proteomics, and biomechanics increasingly reveal the complexity underlying native tissue and organ structure and function. Identifying methods to repair, regenerate, or replace vital tissues and organs remains one of the greatest challenges of modern biomedical engineering, one that deserves our very best effort. Notwithstanding the continuing need for improving standard methods of investigation, including cell, organoid, and tissue culture, biomaterials development and fabrication, animal models, and clinical research, it is increasingly evident that modern computational methods should play increasingly greater roles in advancing the basic science, bioengineering, and clinical application of regenerative medicine. This brief review focuses on the development and application of computational models of tissue and organ mechanobiology and mechanics for purposes of designing tissue engineered constructs and understanding their development *in vitro* and *in situ*. Although the basic approaches are general, for illustrative purposes we describe two recent examples from cardiovascular medicine—tissue engineered heart valves (TEHVs) and tissue engineered vascular grafts (TEVGs)—to highlight current methods of approach as well as continuing needs.

## Introduction

Tissue engineering seeks to repair, regenerate, or replace diseased or damaged tissues and organs using advances in molecular, cellular, and matrix biology as well as biomaterials and bioengineering. Early foundations upon which this growing field rest include work in the late 1970s and early 1980s wherein it was shown *in vitro* that cells can organize reconstituted structural proteins into tissue-like structures.^5^ Indeed, it was suggested soon thereafter that blood vessels could be engineered *in vitro* using collagen tubes seeded with vascular cells.^84^ This early promise has now resulted in a clinical reality, with multiple centers reporting successes of tissue engineered vascular grafts (TEVGs) in patients in Asia, Europe, and the USA.^8,33,51^ Notwithstanding the demonstrated potential and continuing promise of these and related technologies, they developed largely *via* trial-and-error empirical approaches over long periods. There is, therefore, a pressing need both to improve and to accelerate the workflow from concept to clinical utility. In this brief review, we suggest that computational modeling can play important roles in this regard. Toward this end, we illustrate past accomplishments and emphasize future needs primarily *via* two illustrative examples from cardiovascular medicine and surgery—the design and use of tissue engineered heart valves (TEHVs) and TEVGs. Moreover, we focus on how the intrinsic response of living tissues to mechanical stimuli can be leveraged to guide neotissue development and adaptation toward successful outcomes in Regenerative Medicine.

## Foundations: A Brief on Mechanobiology

Many cell types are sensitive to changes in their mechanical environment, often changing gene expression transiently to facilitate adaptive responses. Of particular importance in cardiovascular tissue engineering, endothelial cells are exquisitely sensitive to changes in blood flow-induced wall shear stress. For example, they upregulate their production of nitric oxide in response to increases in wall shear stress and upregulate endothelin-1 in response to decreases in wall shear stress. In both cases, these potent vasoregulatory molecules diffuse into the vascular wall and relax or contract, respectively, the smooth muscle cells that drive the vasodilation or vasoconstriction that is needed to return the wall shear stress toward normal values.^10^ Similarly, smooth muscle cells, fibroblasts, and valve interstitial cells respond to increases in pressure-induced tissue stresses or strains by altering gene expression, often resulting in changes in production (synthesis) and removal (degradation) of extracellular matrix constituents^2,11,25,29^ to return the mechanical state toward normal. It is, of course, the extracellular matrix that endows the tissues and organs of the cardiovascular system with appropriate stiffness and strength, hence mechanical stimuli-driven changes often lead to changes in composition and properties that affect function. As an example, increased cyclic stretching of vascular smooth muscle cells tends to increase local expression of angiotensin II and transforming growth factor-beta,^55^ which through separate but complementary cell signaling pathways (mitogen activated protein kinases in the former, and Smads in the later) drive altered rates of extracellular matrix production and removal, with cyclic stretching also affecting the production, activation, and efficacy of matrix metalloproteinases. Importantly, this mechano-sensing mediated synthesis of matrix is complemented by a mechano-regulated assembly of the newly deposited matrix that depends on actomyosin activity, which allows the cells not only to organize new matrix but also to reorganize extant matrix.^56^ Cross-linking of the matrix *via* lysyl oxidase or transglutaminases, respectively, ensures mechanical functionality.

**Figure 1:**
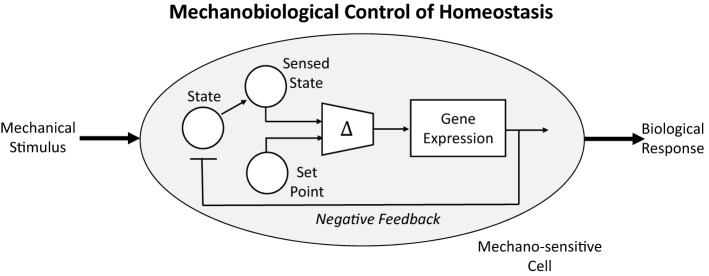
Schema to illustrate mechanobiological control of mechanical homeostasis at the cell level. Mechanical stimuli include applied loads such as blood pressure- and flow-induced stresses or strains. The combination of geometry, material properties, and applied loads define the mechanical state, which can be sensed *via* different receptors including integrins. It appears that the cell compares the sensed state with a preferred homeostatic target, often called a set-point. If the difference Δ between the sensed state and the set-point is within a tolerance, then the cell continues to turnover matrix at balanced basal rates. If this difference is greater than the tolerance, however, then altered and often unbalanced stress- or strain-mediated rates of production and removal in possibly evolving configurations emerge *via* differential gene expression. Homeostasis requires negative feedback, which seeks to maintain or return the state towards its preferred value.

This mechano-control of cell and matrix turnover is consistent with the existence of an underlying mechanical homeostasis (Fig. 1), meaning that such turnover often tends to return particular mechanical quantities (e.g., wall shear stress, tissue stress or strain) toward normal values in response to perturbations in loading.^40^ Importantly, similar processes appear to be operative in tissue engineered constructs, both *in vitro* and *in vivo.*^46^ Conceptually, this homeostatic tendency is illustrated easily for a straight segment of a blood vessel subjected to a steady pressure-driven flow. Consider cell-sensed normalized differences ($\Delta$) in stress from homeostatic values that tend to be minimized *via* a homeostatic response, namely

$$\Delta {\tau }_{w}\equiv \left(\frac{\left(1-\xi \right){\tau }_{w}-{\tau }_{w}^{o}}{{\tau }_{w}^{o}}\right)\to 0, \Delta {\sigma }_{\theta }\equiv \left(\frac{\left(1-\delta \right){\sigma }_{\theta }-{\sigma }_{\theta }^{o}}{{\sigma }_{\theta }^{o}}\right)\to 0,$$

with ${\tau }_{w}$ and ${\sigma }_{\theta }$ the perturbed values of the mean flow-induced wall shear stress and pressure-induced intramural circumferential stress, respectively, where values having a superscript “*o*” denote original homeostatic target values (or set-points). The parameters $\xi \in [\mathrm{0,1}]$ and $\delta \in [\mathrm{0,1}]$ account for the possibility that the cells may not sense exactly the actual value of stress that is dictated mechanically by the geometry, material properties, and applied loads at any instant; that is, cells can only respond to that which they perceive to be differences from normal. In this simple case, if one lets perturbed values of volumetric flow $Q$ and local pressure $P$ be represented by $Q=\varepsilon {Q}_{o}$ and $P=\gamma {P}_{o}$, where $\varepsilon$ and $\gamma$ represent fold-changes from original values (again denoted by index *o*), and if one considers simple steady-state / equilibrium solutions (${\tau }_{w}=4\mu Q/\pi {a}^{3}$ and ${\sigma }_{\theta }=Pa/h$, where $\mu$ is viscosity, $a$ is luminal radius, and $h$ is wall thickness),^38^ then mechanical homeostasis requires specific geometric changes in addition to compositional changes, namely

$$\left(1-\xi \right){\tau }_{w }\sim { \tau }_{w}^{o} \Rightarrow a \sim { \left(1-\xi \right)}^{1/3}{\varepsilon }^{1/3}{a}_{o},$$

and similarly,

$$\left(1-\delta \right){\sigma }_{\theta } \sim { \sigma }_{\theta }^{o} \Rightarrow h \sim { \left(1-\xi \right)}^{1/3}{\varepsilon }^{1/3}(1-\delta )\gamma {h}_{o},$$

which in the case of perfect cell sensing ($\xi =0, \delta =0$) reduces to^36^: $a \sim {\varepsilon }^{1/3}{a}_{o}$ and $h \sim {\varepsilon }^{1/3}\gamma {h}_{o}$. Although these simple results hold only for idealized cases of perturbed blood flow and pressure within a uniform thickness and diameter cylindrical tube, they yet apply conceptually to many specific vascular adaptations and, importantly, they provide valuable insight when growth (changes in mass) and remodeling (changes in microstructure), denoted G&R, are stress mediated. For example, these relations illustrate the need to account for possible changes in the ability of a cell to sense its environment (as in cases of mutations to integrins or their extracellular matrix ligands as well as mutations that alter the actomyosin activity needed to effect sensing), the need to determine homeostatic target values (set-points, which can vary from site to site within the cardiovascular system and from species to species and yet be maintained locally), and the utility of identifying measurable quantities (such as changes in radius and thickness) that can be used to assess the validity of particular hypotheses or computational results. In addition, there remains a pressing need for precise mechanobiological relations (mechanical dose response curves) that allow one to quantify molecular-level changes in response to tissue-level changes in mechanical loads. We discuss below a class of G&R computational models that are informed by such information.

## Foundations: Advances in Computational Modeling

### Finite Element Modeling of Biofluids, Biosolids, and Their Interactions

Computational fluid dynamics (CFD) has emerged as a powerful tool for biomedical analysis and design^81^ and so too computational biosolid mechanics.^58^ For example, specialized codes such as *SimVascular* (simvascular.org) and *CRIMSON* (crimson.software) enable CFD and fluid–solid interaction (FSI) solutions of unsteady, three-dimensional blood flow and pressure within complex, patient-specific domains. These models are also coupled easily with reduced-order models (e.g., lumped parameter networks) to generate computationally efficient multi-domain models of networks of blood vessels, including those within organs. Noting that most soft tissues and organs exhibit material and geometric nonlinearities, it is fortunate that even early advances in finite element analysis (FEA) allowed study of complex nonlinear materials undergoing finite deformations.^67^ Many advances thereafter enabled solutions of complex initial-boundary value problems relevant to biological tissues, again for patient-specific geometric domains. Among others, the specialized code *FEBio* (*febio.org*) is now widely used in biomechanics, though other common commercially available non-specialized codes (e.g., ABAQUS) are used as well.

### Growth and Remodeling (G&R) Models

Notwithstanding the importance of quantifying the complex mechanics experienced at any time by cardiovascular and cardiopulmonary tissues and organs, including their interactions with the flowing blood (cardiovascular) or air (pulmonary), it is the ability of these tissues to grow and remodel during morphogenesis, homeostasis, and pathogenesis in response to diverse stimuli that truly distinguishes them as complex living materials. Such stimuli include the implantation of polymeric materials, which typically elicit a foreign body immune response while also changing the local mechanical environment.

**Figure 2:**
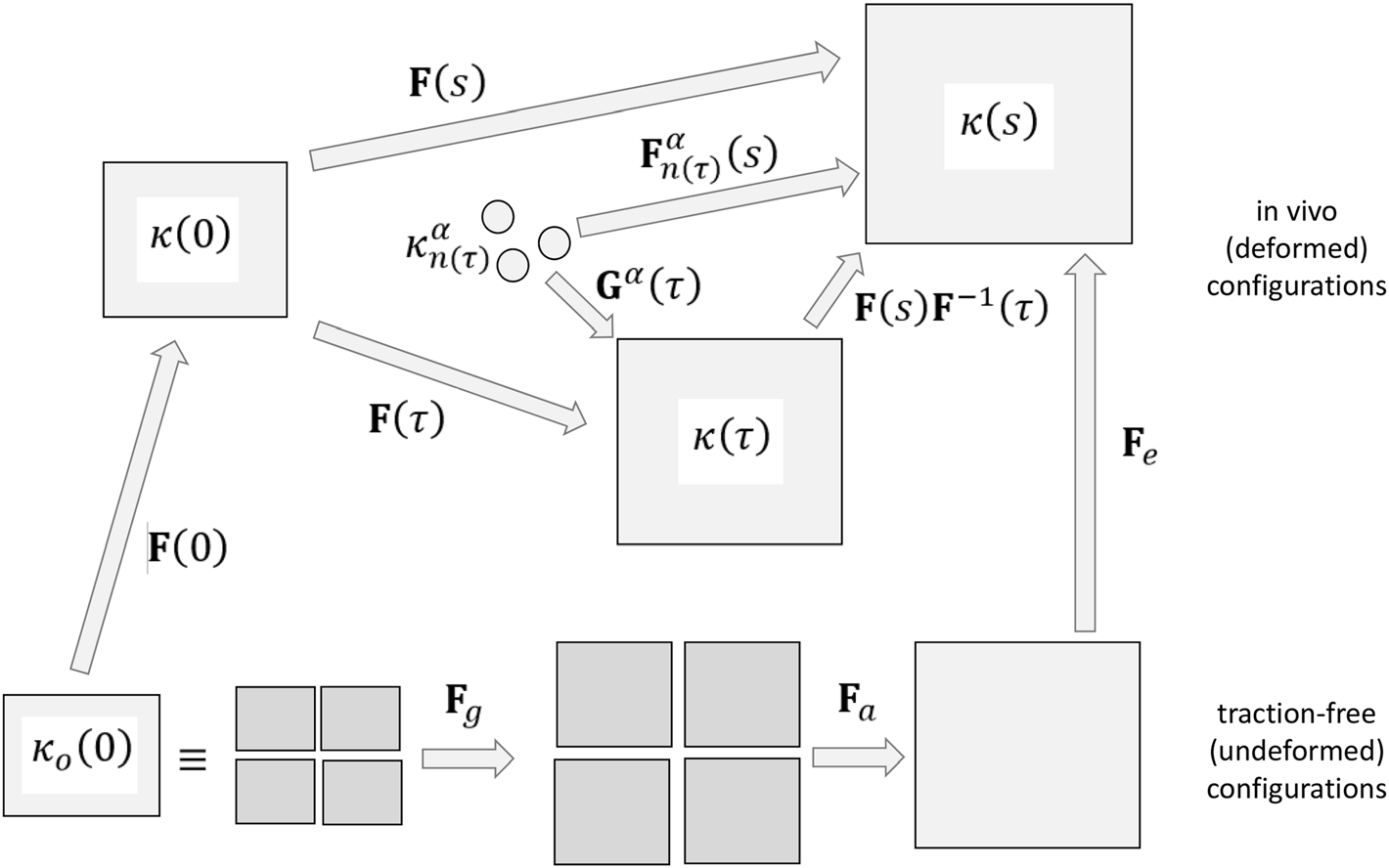
Schematic drawing of motions associated with the two common theories of G&R. In the kinematic growth theory, the deformation gradient of interest (mapping material particles from an original traction-free reference configuration ${\kappa }_{o}\left(0\right)$ to the current loaded configuration $\kappa (s)$) is given by ${\varvec{F}}={\varvec{F}}_{e}{\varvec{F}}_{a}{\varvec{F}}_{g}$ to account for growth (g), then elastic assembly (a), then traction-induced elastic (e) deformations. That is, it is assumed that growth occurs in fictitious stress-free portions of the body, denoted by darker grey filling, that need not be compatible but must be assembled prior to subsequent elastic deformations, with the material behavior given by a $W({\varvec{F}}_{e}{\varvec{F}}_{a})$. By contrast, in the constrained mixture theory it is the deformation experienced by individual constituents $\alpha$ relative to their individual (possibly evolving) natural configurations ${\kappa }_{n(\tau )}^{\alpha }$ that is important, with ${\varvec{F}}_{n\left(\tau \right)}^{\alpha }\left(s\right)={\varvec{F}}(s){\varvec{F}}^{-1}(\tau ){\varvec{G}}^{\alpha }(s)$ determined *via* finite deformations experienced by the tissue (mixture) plus constituent-specific deposition stretches ${\varvec{G}}^{\alpha }(s)$ that pre-stress newly deposited matrix; note that G&R time $\tau \in [0,s]$. The material behavior of individual constituents is then given by a mass-averaged ${\widehat{W}}^{\alpha }({\varvec{F}}_{n\left(\tau \right)}^{\alpha }\left(s\right))$. Clearly, both theories include multiplicative finite deformations and specialized tensors, ${\varvec{F}}_{g}$ or ${\varvec{G}}^{\alpha }(s)$, which must be prescribed constitutively. Although relations can be derived to relate the different kinematics, there is no advantage in doing so. The approaches are conceptually different, with the kinematic growth theory focusing on growth of fictitious stress-free configurations and the constrained mixture approach focusing on constituent-specific deformations of individually deposited constituents within *in vivo* configurations.

Two basic approaches have emerged for modeling G&R of soft tissues and organs: the theory of kinematic growth^74^ and the constrained mixture theory.^39^ Briefly, in the kinematic growth approach, one writes the finite deformation gradient in terms of a multiplicative decomposition, with one part described by a growth tensor and one described by a traditional (elastic) deformation gradient (Fig. 2). In the original approach, the growth tensor describes the evolution of fictitious stress-free configurations during G&R, often using a homeostatic assumption that such growth is driven by differences in a target quantity (often stress) from its homeostatic set-point. The elastic deformation gradient can include two successive motions: one from stress-free configurations that emerge during growth to an assembled traction-free reference configuration that is often residually stressed and another from the assembled reference configuration to an *in vivo* loaded configuration. This general approach has been used widely, in part due to the computational ease of calculations.^1,80^

By contrast, the constrained mixture theory focuses primarily on mass balance, not deformations. Briefly, a full mixture relation for mass balance for $\alpha$=1,2,3,…,*N* structurally significant constituents reveals the need for *N* constitutive relations for mass exchange, which in a rate-based formulation is simply the difference between constituent-specific true mass density production (e.g., synthesis, proliferation) and removal (e.g., degradation, apoptosis) functions. Under the assumption that the many structurally significant constituents may possess different (evolving) natural configurations while yet being constrained to move together with the mixture, and that such motions are slow in G&R processes at the tissue level relative to cyclic applied loads, the mixture mass balance equation can be integrated directly to yield a heredity integral (fading memory) based formulation in terms of the true mass density production function ${m}^{\alpha }\left(s\right)>0$ and an associated survival function ${q}^{\alpha }\left(s-\tau \right)\in [{0,1}]$. Here, $\tau \in [0,s]$ is the G&R time at which constituent $\alpha$ was deposited within extant matrix and $s$ is the current G&R time. This survival function can capture, for example, the different finite half-lives exhibited by different cells and matrix constituents under different conditions. These relations for mass balance have been used to motivate *N* constituent-specific constitutive relations for stored energy, which when summed yield the total stored energy that is needed to solve initial-boundary value problems either in weak (finite element) or strong (analytical) form, though typically with considerable computational expense. For this reason, multiple simplifying assumptions have been invoked to yield computationally less expensive formulations of a constrained mixture, including so-called homogenized^12^ and mechanobiologically equilibrated^53^ formulations. Similar to the kinematic growth models, constituent-specific finite deformations in the constrained mixture models involve multiplicative decompositions (Fig. 2), though with a fundamental role played by an internal variable called a deposition stretch tensor that prescribes the mechano-regulated pre-stretch (pre-stress) under which new matrix is incorporated within extant matrix.

Constitutive relations in constrained mixture models often describe cell and tissue production and removal in terms of differences in scalar measures of stress or strain from homeostatic target values (recall Fig. 1), not unlike for the homogenized growth tensor in kinematic growth models. For example, recalling the aforementioned relations for differences in stress from normal values, illustrative production and removal terms can be written, respectively, for a blood vessel as

$${m}^{\alpha }\left(s\right)={m}_{o}^{\alpha }(1+{K}_{\sigma }^{\alpha }\Delta \sigma -{K}_{{\tau }_{w}}^{\alpha }\Delta {\tau }_{w}+\cdots )$$

and, within the context of a generalized first-order decay,

$${q}^{\alpha }\left(s,\tau \right)=exp\left(-{\int }_{\tau }^{s}{k}_{o}^{\alpha }\left(1+\omega \Delta {\sigma }^{2}+\cdots \right)dt\right)$$

where ${m}_{o}^{\alpha }$ are constituent-specific basal rates of mass production, ${K}_{i}^{\alpha }$ are constituent-specific gain parameters, and ${k}_{o}^{\alpha }$ are constituent-specific basal rate parameters. Note that the first relation shows that rates of production can be modulated, depending on the gain-dependent sensitivity of the cell to the particular difference from normal, by multiple stimuli: the positive sign for the intramural stress stimulus accounts for increases in production when the sensed stress rises above its target value(s) whereas the negative sign for the shear stress accounts for nitric oxide slowing the rate of matrix production (when shear stress rises above its target value) and endothelin-1 heightening it (when shear stress drops below its target value). Note, too, that these relations in terms of gain parameters represent a type of proportional negative feedback control consistent with the concept of homeostasis, with *homeo* meaning “similar to” in contrast to *homo*, which means “the same as.” Finally, for these relations, note that mechanical homeostasis requires ${m}_{o}^{\alpha }={\rho }_{o}^{\alpha }{k}_{o}^{\alpha }$, which is to say that rates of production and removal must balance within the (mechanically unchanging) homeostatic state, with ${\rho }_{o}^{\alpha }$ the constituent-specific apparent mass densities in that state.

Of particular importance herein, mixture formulations can easily account for the different mechanical properties and degradation kinetics of polymeric scaffolds and the deposited neotissue, whether within a bioreactor (*in vitro*) or within the intended location within the body (*in situ*). For additional details on these two methods of modeling G&R, the reader is referred to the following.^1,37,80^

### Fluid–Solid-Growth (FSG) Models

Melding FSI and G&R models leads naturally to FSG models, which are ultimately needed to address most problems within the cardiovascular system. Although the need for and feasibility of these FSG models was established more than a decade ago,^19,41^ and various implementations have arisen since,^27,63^ the challenge of building such computational models remains considerable. Of particular note in the cardiovascular system, the time scale for FSI simulations is on the order of a heart beat (< 1 s) to resolve hemodynamics over a cardiac cycle whereas G&R simulations are typically on the order of days to weeks to months. These disparate times scales typically necessitate loose coupling (handshaking) schemes, ones that should be adaptive because periods of rapid G&R can be followed by a period of slower G&R. Whereas such FSG models are discussed further below, note that direct coupling of hemodynamics and G&R can be achieved using reduced-order models for the blood flow, as, for example, a control volume relation based on energy balance.^54^

To render these general concepts more tangible, in the following two sections, we consider two illustrative examples of computer model-based design and analysis of tissue engineered constructs, one that led to new insight into design requirements and one that aided clinical management.

## Illustration 1: Tissue Engineered Heart Valves (TEHVs)

### Clinical Relevance

Current treatment options for end-stage valvular heart disease are largely limited to surgical replacement of the valve with either a mechanical or biological (e.g., chemically fixed xenograft) valve substitute.^20^ Both types of prostheses are non-living, and thus cannot grow, adapt, or repair when needed. This lack of adaptive capacity often leads to complications (e.g. limited durability or size mismatch due to patient growth) that require repeat surgeries. This deficiency translates into a significantly reduced life expectancy of recipients of valve replacements, particularly for young patients.^32,50,69^ By contrast, TEHVs are living tissues having regenerative capacity, which thereby have the potential to become superior valve replacements compared to contemporary valve replacements.^42,43^ To enable this transition, the regeneration and adaptation of TEHVs should be understood and well controlled such that proper, ideally native-like, valve function is established during tissue development and maintained over long periods through tissue adaptation and repair.

### Approaches and Challenges

The basic principle of heart valve tissue engineering is that a non-living, biodegradable, valve-shaped scaffold is infiltrated by cells, which subsequently transform the degrading scaffold into a living tissue having growth and remodeling capacity. Traditionally, TEHVs were created *in vitro* using bioreactor platforms before implantation. Both decellularized biological matrices and biocompatible, biodegradable synthetic materials have been used as scaffold materials. Decellularized allografts and xenografts benefit from a more native-like tissue architecture compared to synthetic materials, and promising clinical outcomes have been reported.^9,14^ Yet, concerns remain regarding limited cell infiltration into biological matrices, the limited availability of allografts, and the zoonotic risks associated with xenografts. Synthetic scaffolds offer improved control over material properties and allow more rapid cell infiltration compared to biological materials. Nevertheless, despite promising early results,^34,61,76^ long-term pre-clinical studies repeatedly report progressive leaflet retraction and thickening due to cell-mediated tissue remodeling after implantation.^22,24,73^

Due to the scientific and regulatory complexity associated with the implantation of living tissues, current efforts are now focused mainly on *in situ* heart valve tissue engineering. In the *in situ* approach, non-living biodegradable valve-shaped scaffolds are implanted directly at the functional site, where they gradually transform into living heart valves. Despite promising results, regeneration of *in situ* TEHVs has so far been unpredictable and variable, which restricts their safe clinical translation. Specifically, substantial spatial differences in tissue formation and scaffold resorption have been observed, potentially correlated with spatial differences in cell infiltration as well as mechanical stimuli.^6,13,21,57,82^ Similar to the *in vitro* approach, major complications include progressive leaflet retraction and thickening, which ultimately lead to a loss of valve functionality. Additionally, the potential for functional growth of *in situ* TEHVs has yet to be demonstrated.

### Contributions of Computational Models

The main issues underlying many of these challenges are a poor understanding of progressive tissue regeneration and adaptation as well as limited predictive capabilities. We submit that computational models, particularly when integrated with experimental measurements, can help to overcome these issues. For example, computational models have been developed to understand how mechanical stresses and strains direct collagen remodeling in heart valves,^3,4,16^ how (oscillatory) fluid flow and deformation influence *in vitro* engineered tissue formation,^71,72,75^ and how cellular traction forces and collagen remodeling can lead to leaflet retraction.^57^ Ultimately, mechanistic computational models of tissue regeneration and adaptation should be combined with optimization algorithms to efficiently identify promising scaffold designs, ideally as a function of patient-specific parameters that drive the regeneration, functionality, and adaptative capacity of TEHVs. Such models promise to accelerate the clinical translation of TEHVs and to reduce the number of animal experiments since only the most promising TEHV designs need to be tested experimentally.

As an example, we recently showed that computational models can enable breakthroughs in improving TEHV adaptation and functionality. For this, we focused on understanding and eliminating the development of progressive leaflet retraction in *in situ* TEHVs, as this has been one of the most common failure modes of TEHVs over the past 10–15 years.^17,22,24,73,78^ Computational analysis of *in vivo* leaflet strains as a function of valve geometry and collagen anisotropy^57^ suggested that the often-used Thubrikar design was prone to adverse tissue remodeling leading to leaflet retraction in TEHVs. A more curved valve geometry was predicted computationally to yield more favorable leaflet strains with respect to valve functionality, and we hypothesized that this could also improve the strain-mediated remodeling of the TEHV. We subsequently investigated *in situ* the remodeling of TEHVs featuring this more curved leaflet geometry by implanting them as pulmonary valve replacements in sheep for 1 year. The results of this pre-clinical study confirmed substantially reduced leaflet retraction and preserved functionality of most TEHVs throughout the complete follow-up period.^18^ Moreover, our computational models were able to predict the *in situ* remodeling of these TEHVs from the initial tissue properties at implantation, thus explaining differences in outcome due to variations in tissue properties or hemodynamic loading conditions. Altogether, this example demonstrates that computational models can significantly advance our understanding of TEHV remodeling, predict *in vivo* outcomes, and reveal novel design strategies for improving the adaptation and corresponding functionality of TEHVs.

## Illustration 2: Tissue Engineered Vascular Grafts (TEVGs)

### Clinical Relevance

Disease and injury are responsible for many clinical interventions designed to support, repair, or replace blood vessels. Examples include use of prosthetic grafts in the repair of an aneurysmal aorta, use of autologous vein grafts in coronary artery bypass surgery, and use of prosthetic grafts in the Fontan completion surgery for palliative treatment of children with congenital heart defects. Grossly damaged blood vessels resulting from traumatic injury can similarly be replaced or supported by such grafts, and shunts can be introduced surgically to facilitate important clinical care, including hemodialysis. Despite the life-saving emergence of standard-of-care vascular grafts fabricated from Dacron (poly(ethylene terephthalate), a polyester) or GoreTex™ (poly(tetrafluoroethylene), or PTFE), synthetic grafts yet associate with complications and failures that are responsible for continuing morbidity and mortality. Such complications include infections and thrombosis, but also longer term dilatation or stenosis, the latter sometimes including calcification. Indeed, thrombosis remains a continuing barrier to the use of synthetic grafts in the case of small vessels. A particular limitation of synthetic grafts in pediatric applications is their lack of growth potential. For these and other reasons, many have turned to the promise of tissue engineering in the search for improved vascular conduits, particularly since publication of the seminal paper by Dr. Laura Niklason and colleagues more than 20 years ago.^64^

### Approaches and Challenges

As in the development of TEHVs, there are two basic approaches to constructing TEVGs: *in vitro* development in specially designed bioreactors and *in situ* development at the intended site within the body. Regardless, the basic requirement is again that cells must infiltrate an initial scaffold and begin to form neotissue *via* deposition of extracellular matrix constituents. These initial scaffolds can consist of biodegradable polymers, such as poly(glycolic acid) and poly(ε-caprolactone), reconstituted extracellular matrix, often type I collagen or fibrin, or decellularized native vessels in the hope of retaining much of the complexity of normal tissues. A native blood vessel consists primarily of elastic fibers (i.e., elastin and elastin-associated glycoproteins such as fibrillin-1) and fibrillar collagens (especially types I and III) in addition to three primary cell types (endothelial, smooth muscle, and fibroblasts), but nevertheless contains ~ 100 different proteins, glycoproteins, and glycosaminoglycans.^47^ It is unimaginable that such matrix complexity could be reproduced in the laboratory, hence one seeks to use cells to produce and organize the matrix in which they need to reside *in vivo*.

### Contributions of Computational Models

We and others have used computational models to advance the development of TEVGs. Noting that vessel-level compliance depends on both the geometry and intrinsic material properties of the TEVG, Vande Geest and colleagues used computational methods to design and fabricate compliance-matching grafts.^30^ In particular, they first modulated gelatin-based, fibrinogen-mediated electrospun tubular constructs to mimic the compliance of the abdominal aorta in a rat interposition model. The intrinsic stiffness of the fibers was altered *via* different periods of cross-linking (2, 8, and 24 h), with interpolated values used to identify that period of cross-linking that yielded the desired overall compliance given near aorta-matching diameter and wall thickness. A subsequent study showed that a similar computational identification of a TEVG having a preferred compliance at implant yielded excellent short-term (4 weeks) *in vivo* results.^23^

Conversely, our group developed a computational G&R model of both *in vitro* and *in situ* TEVG development,^59,65^ the latter based on extensive data from a mouse model developed by Dr. Christopher K. Breuer. Briefly, for the mouse model, the small diameter scaffold consisted of a tubular poly(glycolic acid) felt with a 50–50 co-polymer sealant of poly(ε-caprolactone and L-lactide). The scaffolds were implanted as interposition grafts in the inferior vena cava of mice, some immuno-competent and some immuno-compromised to study immune factors, to simulate the lower blood pressures in the Fontan conduit (relative to systemic pressures). The vessels were imaged longitudinally *in vivo* and were harvested at times ranging from two weeks to two years, then subjected to biomechanical testing and immuno-histological examinations. The histo-mechanical data were then used to inform a constrained mixture based G&R model, which accounted for the geometry and mechanical properties of the initial polymeric scaffold and its degradation profile as well as subsequent cell-mediated deposition and degradation of collagen-dominated neotissue. Because of the initial strong foreign body response, largely by macrophages,^70^ the mass production term ${m}^{\alpha }(s)$ was both immuno-driven and mechano-mediated, with the latter accounting for initial stress shielding of the infiltrating synthetic cells by a stiff polymeric scaffold that eventually gave way to normal mechano-sensing. Parameterization of the model provided good descriptions of the evolution of the mechanical properties of the TEVG over a 6-month period of implantation,^59^ which was found to predict well the evolution over a subsequent 18-month period.^48^ Importantly, this basic model also predicted a narrowing of the graft that should resolve spontaneously over a period of weeks; that is, an early exuberant immuno-driven production of matrix thickened the wall, which encroached on the lumen, but then reversed as the inflammation waned with polymer degradation. This unexpected computational prediction was validated in a pre-clinical sheep model over an 18-month study period, which confirmed an early narrowing of the TEVG (by 6 weeks of implantation) with a subsequent, spontaneous resolution of the narrowing within 18 weeks.^15^ This important finding resolved a previously identified clinical concern and thus enabled US Food and Drug Administration (FDA) approval of this promising technology for use as a Fontan conduit in children with congenital heart defects, which is now in clinical trials. In particular, consistent with model predictions, the patients are now followed more closely with medical imaging and interventional angioplasty is reserved for only symptomatic narrowing. Hence, computational models can provide increased insight into TEVG development and help to guide clinical management.

Validated G&R models of *in situ* development of TEVGs promise yet another important application, rational design of the scaffold itself. Coupling methods of non-dimensionalization with micromechanical analyses of the properties of porous scaffold materials allowed parametric simulations of the *in situ* evolution of material properties of a TEVG as a function of the initial polymeric scaffold pore size, fiber diameter, and porosity.^60^ These simulations revealed interesting, initially non-intuitive results, including that an initially stiff, low-porosity scaffold could yield a more compliant neovessel at late times since an initial low porosity reduces early inflammatory cell infiltration and thus attenuates the immuno-driven deposition of matrix that is expected due to the implantation of a foreign body. It was later shown that formal methods of optimization, including use of the non-intrusive, derivative-free surrogate management framework, could be used to identify preferred values of particular scaffold parameters based on simulated long-term *in situ* development.^79^ Yet, as one would expect, the predicted optimization of the *in situ* development depended strongly on the objective function used, noting that there are many parameters to optimize, including initial scaffold dimensions, microstructure, and overall compliance. Perhaps more importantly, for the polymers simulated, it was clear that the high stiffness of these materials relative to native makes it very difficult to design an initial scaffold having appropriate compliance.

## Discussion and Future Directions

**Figure 3:**
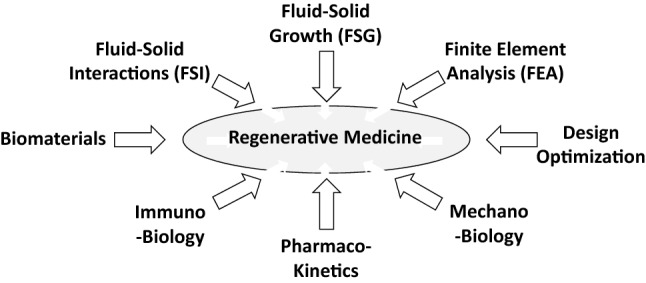
Schema to illustrate some of the many different fields and approaches that contribute to the design of safe and effective regenerative medicine solutions. Such work necessarily builds upon the basic cell and matrix biology, here noted specifically in terms of the immuno-biology and the mechano-biology. Biomaterials, both synthesis and fabrication, similarly plays a central role as should modern methods of design, including formal methods of optimization. Finally, we submit that computational models necessarily must play an increasing role in the conceptualization, design, fabrication, and clinical management of regenerative medicine technologies, to include in the cardiovascular system fluid–solid-interaction (FSI) solutions and finite element analysis (FEA). To these one should add computational models of tissue growth and remodeling (G&R), noting that coupling FSI + G&R yields novel fluid–solid-growth (FSG) simulations, which should be multiscale and include a coupling of cell or cell-signaling models. Finally, one should consider particular adjuvant pharmacotherapies, which should be designed using computational solutions of the associated kinetics. Although other areas can contribute, these eight should be considered fundamental and integrative.

Much has been achieved, yet much remains to be accomplished for Regenerative Medicine to achieve its full potential and become a consistent contributor to improving human health-span and life-span.^7,28,83^ Toward this end, there is a need for advances along all fronts: a better understanding of the associated molecular and cell biology in native and diseased tissues and organs, development of advanced biomaterials that can better immuno-modulate the host response, perhaps *via* addition of biomimetic materials, and of particular importance here, advances in computational models that can be used to inform design and to predict outcomes. Computational models of the associated molecular and cell biology could also help identify short-term adjuvant pharmacotherapies,^49^ and so too computational models of the pharmacokinetics. See, for example, Fig. 3 which highlights a few of the many areas of biology, biomaterials, and bioengineering that have contributed, and must continue to contribute, to advancing Regenerative Medicine. Of particular note herein, the general promise of computational modeling has been recognized and supported by the US FDA as an efficient approach to advance biomedical device design and development^62^. In Europe, roadmaps have been defined toward realizing the Digital Patient (DISCIPULUS project, https://www.vph-institute.org/discipulus.html) and defining strategies for *in silico* clinical trials (AVICENNA project, https://www.vph-institute.org/avicenna.html).

Fortunately, fabrication methods for building tissue engineering scaffolds have also advanced tremendously, now including traditional methods used to make textiles as well as electro-writing and 3D (bio)printing. With these advances comes the ability to control many different design parameters,^45,66,77,85^ including chemical composition, porosity, fiber diameter and alignment, pore size, and degradation profile as well as graft-level geometry (layered or not), compliance, and strength. As noted above, much of scaffold design yet remains empirical, seeking systematically to control many of the key variables of interest. Computational models promise to reduce the parameter search space,^26,35^ thus reducing the experimental need and potentially accelerating the time to pre-clinical testing. Yet, to fully leverage the potential of computational models in Regenerative Medicine, several challenges still need to be addressed in the future.

### Multiphysics Models

Tissue engineering scaffolds must be designed with the end in mind—effective performance over long periods *in vivo*, which may necessitate appropriate growth and adaptive capabilities in response to potentially changing loads. In many ways, the cardiovascular system serves well as an archetype for the need for multiphysics modeling to describe a complex *in vivo* environment. The primary function of the cardiovascular system is biotransport, leading to the transfer of nutrients, gases (O_2_ and CO_2_), and waste products to and from tissues throughout the body. Towards this end, complex nonlinear biosolid tissues facilitate the pressure-driven flow of a complex biofluid throughout the body. Hence, comprehensive models need to account for fluid–solid interactions that result in effective mass, momentum, and heat transfer. As noted above, FSG models^19^ thus become important because these tissues can grow and remodel (adapt) in response to changes in loading. There remains a pressing need to identify and implement an efficient FSG model.

### Multiscale Models

There continues to be a pressing need for computational biomechanical models from transcript-to-tissue, particularly given recent advances in transcriptomics and proteomics. In particular, although most engineered tissues and organs must function at a macroscale, it is the molecular mechanisms at the microscale that ultimately dictate success or failure. As noted earlier, even changes to mechanical loading in native tissues and organs drive myriad differentially expressed genes (DEGs). Multiscale models can and should account for tissue-level phenomena (continuum biomechanics), constituent-level mechanisms (e.g., turnover of particular structurally significant constituents *via* G&R mechanics), and cell-level behaviors (e.g., migration, proliferation, apoptosis, and differentiation) that are characterized by transcriptional changes (driving changes in gene products). The aforementioned constrained mixture G&R formulations naturally meld the continuum- and constituent-level behaviors, though there remains a need for advances in both constitutive formulations and computational implementations.^37^ Additionally, it has been shown that these same constrained mixture models can be linked with cellular automaton (e.g., agent based) models to account for cell-level behaviors^31^ and recently it has been shown that constrained mixture models can similarly be linked with logic-based cell signaling models, which enable transcriptional changes to affect tissue level responses.^44^ Thus, it is clear that true multiscale—tissue to constituent to cell to cell signaling—models can be built, and even coupled with hemodynamic models, yet the current implementations remain in their infancy.

### Machine Learning Models

Advances in scientific machine learning continue to emerge rapidly, with considerable promise to advance biomedical research and clinical care. With regard to Regenerative Medicine, there is an opportunity both to augment scaffold design and extend predictions of the *in situ* development of tissue engineered constructs. Indeed, fundamental to the continuing success of tissue engineering is improved methods of scaffold design. Given the broad design space, defined by so many free parameters, machine learning methods that enable identification of preferred microstructural properties offer considerable promise.^86^ Machine learning models can also complement the multiscale, multiphysics models^1,68^ that will be fundamental to advancing tissue engineering.

## Conclusion

In summary, we used two illustrative examples from cardiovascular medicine and surgery—TEHVs and TEVGs—to illustrate promising roles of computational modeling in understanding and advancing Regenerative Medicine. There are, of course, many other examples, including the earlier use of computational modeling to design scaffolds for bone regeneration.^35^ With regard to the future, we expect that computational models will play increasingly important roles in advancing tissue engineering in particular and Regenerative Medicine in general.
